# The Role of Academic Resilience, Motivational Intensity and Their Relationship in EFL Learners' Academic Achievement

**DOI:** 10.3389/fpsyg.2021.823537

**Published:** 2022-01-26

**Authors:** Shengli Yang, Weirong Wang

**Affiliations:** School of Economics, Hebei University, Baoding, China

**Keywords:** academic resilience, positive psychology, academic achievement, language educational system, motivational intensity

## Abstract

The aim of developing academic resilience and motivational intensity, as two constructs of positive psychology, is to increase learners' capability to compete with each other even in adverse conditions. Different types of academic resilience are conceptualized and germane literature about the relationship between academic resilience and academic achievement is provided. Literature showed that some socio-affective factors (e.g. peer relations, parents' high expectations, teachers' attention, and kindness, etc.), socio-economic factors (e.g. the financial contribution of parents' to education, economic and social class level, etc.), and affective factors (e.g. anxiety, self-efficacy, motivation and so on) can influence learners' academic achievement and policy makers' decision in providing an appropriate context for learning. In the end, the pedagogical implications are expounded to foster the language learning quality and to develop a language educational system. Suggestions for further research are provided to develop the existent literature on the relationship between academic, motivational intensity, and learners' academic achievement.

## Introduction

Todays' life is full of challenges and threats. If people do not equip themselves with the necessary abilities, they will face many problems. In the 21st century, a group of psychologists has realized that man must spend his mental energy on the positive aspects of his experience (Wang et al., 2021). Thus, one of the topics attracting a lot of attention in recent decades is factors rooted in positive psychology (Dewaele et al., 2019). Seligman and Csikszentmihalyi (2000) supported the alternation in the focus of psychology by solving undesirable and challenging life issues to improve constructive qualities. Peterson (2006, p. 4) described positive psychology as “the scientific study of what goes right in life, from birth to death and at all stops in between.” This view emphasizes the capabilities of the individual and the purpose of psychology, based on the tenets of positivism, is to improve the standards of living and to realize latent talents (Seligman and Csikszentmihalyi, 2014). Resilience, as one of the typical notions and constructs, is considered by positive psychology and refers to successful adaptation, despite challenges and threats (Fletcher and Sarkar, 2013). Resilience has a special place in the fields of evolutionary psychology and mental health, so that the number of researches related to this structure is increasing every day (Toland and Carrigan, 2011). Although stressful events have key roles in the occurrence of psychological disorders, subsequent investigations have shown that there are moderating features between demanding events and psychological disorders that cause the effect of events on individuals to be different.

Nowadays, education is comprised of learner-oriented approaches. Such approaches aim to develop practices and skills which prepare learners for independent problem-solving and lifelong learning. Williams and Burden (1997) pointed out that “it is undoubtedly true that learners bring many individual characteristics to the learning process which will affect both the way in which they learn and the outcomes of that process” (p. 88), therefore learning achievement can be ascribed to learner-centered differences between these factors e.g., motivation, resilience, anxiety, willingness to communicate, and so on) or interactions of the factors (Schweisfurth, 2011). These learner individual difference variables can change and individualize the general course of the language learning procedures and these individualized variables can distinguish learners by degree (Dörnyei, 2005). Also, investigators have shown that learner-centered differences are good predictors of foreign language learning achievement (Dörnyei, 2014). This review is going to ponder on two individualized variables in language learning contexts: resilience and motivation.

## Review of Literature

### The Concept of Resilience

According to Rajendran and Videka (2006) resilience is an individual's competence in spite of noticeable stressors. In other words, resilience, in the educational context, refers to the learner's ability to achieve his/her objectives while confronting unfavorable or distressing situations (Edwards et al., 2016). Truebridge (2016) described resilience as a dynamic process: “the dynamic and negotiated process within individuals (internal) and between individuals and their environments (external) for the resources and supports to adapt and define themselves as healthy amid adversity, threat, trauma, and/or everyday stress” (p. 15).

Howe et al. (2012) described resilience from two different viewpoints. They provided the psychological definition of resilience based on which “resilience is a dynamic process encompassing positive adaptation within the context of significant adversity” (p. 543). Resilience as a psychological trait can be employed as “a measure of successful stress-coping ability” (Connor and Davidson, 2003, p. 77). Howe et al. (2012) also argued that resilience, from a sociological perspective, can be endorsed or challenged by individual, social and environmental factors. From a social perspective, resilience is theorized as a number of social indices which signify the way of interaction between individuals and their contexts and the kind of chances and occasions that people may have to develop (Ungar, 2012).

Herrman et al. (2011) argued that resilient learners can overcome stress and retain high mental strength regardless of difficulties. Resilience is a combination of psychological and social behaviors as types of dynamic factors (Shin et al., 2009). It can help people to positively function and become accustomed to life tasks within adverse or upsetting situations (Masten and Obradovic, 2006). It can be regarded as a significant individual difference feature in explaining the outperformance of some individuals over others when facing problems (White et al., 2010). Jowkar et al. (2014) argued that resilience can be categorized into behavioral, emotional, and academic groups and the study of academic resilience has drawn the attention of numerous educational investigators.

### Academic Resilience

Resilience, as a motivational-affective variable, has a critical role in the academic field. Current studies have concentrated on distinctive psychological concepts to decrease foreign language learning demotivation and to change L2 learning behavior. Therefore, academic resilience has received renewed attention in the field of foreign learning (Kim and Kim, 2016). Morales and Trotman (2004) mentioned that academic resilience refers to the type of academic attainment regardless of hurdles and hardships. They maintained that academic resilience can be described as the dynamic process through which academically successful individuals can overcome the problems stopping their peers from being successful (Morales and Trotman, 2004). Therefore, academic resilience is determined as both an incentive for attaining academic and individualized objectives, and a provider of adequate mechanisms to cope with stress and nervousness that happen in the University context (Cassidy, 2016).

Knight (2007) argued that highlighting the flaws of learners against the hardships and attempting to discover the solutions are not enough in educational contexts. He mentioned that resilience investigators have radically altered their methods by training and improving the strong points of individuals and they also try to know the factors affecting the resilience of learners to the negative life occurrences. Krovetz (2008) contended that resilience as a multidimensional and developmental process is influenced by individual behaviors, the psychosocial and socio-cultural situations. Consequently, schools and universities are responsible for developing learners' capacities and building up their resilience to be successful (Thomsen, 2002).

### Academic Resilience Factors

Academic resilience has drawn the attention of investigators who inspect the methods of academic achievement for learners and meticulously notice and examine their cognitive and affective procedures. The definition of achievement has mainly broadened and altered. In other words, the achievement is elucidated depending on learners' efficient handling of their cognitive capabilities, self-regulation, and autonomy skills (Schunk and Zimmerman, 2007). Individuals face various reasons or risk factors in their academic life including suffering from chronic disease, living with parents, undergoing a natural disaster, living in financial difficulty, etc. (Masten, 1994). Although these risk factors produce undesirable consequences in a learner's academic life, they can also affect their advancement in numerous fields, and may simultaneously influence the learner's advancement by triggering each other. Furthermore, each learner may be affected differently by these results (Little et al., 2004).

Despite the risk factors, some other factors, called protective ones, are significant in academically resilient learner's accomplishments and they result in positive adaptation (Rojas, 2015). Thus, protective factors, including the quality of learner's characteristics, social context or his/her interaction, and so on are critical to be examined. Recognizing and specifying protective factors help learners save themselves in uncertainties surrounded by their academic life. Poulou (2007) argued that learners' protective factors have a buffer role against the undesirable impacts of hardships lessening the association between risk factors and consequences. Masten and Tellegen (2012) argued that protective factors contribute to the development of positive effects for learners and they categorized these factors into internal and external protective factors. Internal protective factors are associated with person's character whereas external ones are associated with one's social context (Foster, 2013). Fallon (2010) asserted that internal factors including self-efficacy and optimism are the learners' positive attitudes toward adversity. Poulou (2007), on the other hand, defined external factors as the learners' social protection and support against the hardships. For example, a helpful teacher, supportive parents, a society that promotes a secure identity, etc. can be declared as external protective issues.

### The Studies Related to Academic Resilience and Academic Achievement

Some variables in the domain of positivist psychology construct such as emotional regulation, self-compassion and psychological well-being (Rajabi and Ghezelsefloo, 2020; Greenier et al., 2021), burnout, reflection, self-efficacy (Fathi et al., 2021), teacher resilience (Derakhshan, 2021a), academic engagement (Derakhshan, 2021b), and teacher immediacy (Liu, 2021) were investigated. Regarding resilience as a component of positive psychology, some investigations have been carried out. Kim and Kim (2016) studied secondary EFL learners' learning-related resilience by analyzing their perceived happiness, sympathy, sociability, perseverance, and self-regulation. They found out that perseverance and perceived happiness were recognized as the main explanatory factors for EFL learning. Moreover, Proietti Ergün and Dewaele's (Proietti Ergün and Dewaele, 2021) study revealed that resilience is significantly related to teaching enjoyment.

Martin (2013) investigated the academic resilience and buoyancy among Australian high school learners. His study indicated that academic resilience and buoyancy are two separate factors and academic resilience significantly predicted major negative outcomes in learning contexts. Nguyen et al. (2016) study also demonstrated a significant correlation between L2 learners' resilience and experience of storytelling for increasing resilience. They also pointed out that some protective factors for resilience include problem-solving skills, social competence, purpose, use of storytelling for improving resilience and autonomy.

The correlation between academic resilience and academic success has been confirmed in recent studies (Fallon, 2010; Kotzé and Kleynhans, 2013; Kwek et al., 2013; Çelik et al., 2014; Mwangi et al., 2015; Haibin, 2017; Marie et al., 2021). Fallon (2010) investigated 150 Latino low socio-economic class high school learners' academic achievements and their relationship with their academic resilience. The variables including learners' academic achievement, parental involvement, school engagement, and their overall academic resiliency were measured. He found a significant and positive correlation among learners' academic resilience, academic optimism, and academic achievements. Kwek et al. (2013) also found out academic achievement is significantly predicted by self-esteem and academic resilience among Hospitality and Tourism Undergraduate learners. In a study among African junior students, Kotzé and Kleynhans (2013) specified that academic performance is significantly predicted by academic resilience. Çelik et al. (2014) studied the relationships among academic resilience, self-esteem, locus of control, hope and academic achievement among 1,169 Turkish EFL learners. They found out that personal issues, family support, and environmental context, as protective factors, significantly affected developing hope, confidence, academic resilience and academic achievement among learners. Mwangi et al.'s (Mwangi et al., 2015) study also revealed a significant correlation between academic resilience and academic achievement. Haibin (2017) found out that participation in school, supervision of parents, and expectation of school are significant factors in decreasing learners' problematic behaviors and improving their academic resilience and achievement.

de la Fuente et al. (2017) examined the relationship between academic resilience, strategies in facing with academic stress, learning approaches and academic achievement. Using the structural equation model, they found a strong and significant correlation among resilience, deep learning approach and academic achievement. In line with de la Fuente et al.'s (de la Fuente et al., 2017) study, Rodríguez-Fernández et al. (2018) supported the predictive power of academic resilience over linguistic performance among Spanish learners. Karabiyik (2020) also found out that adaptive help-seeking and reflecting, as two components of academic resilience, significantly predicted learners' Grade Point Average (GPA) as measure of academic achievement. Ayala and Manzano (2014) also asserted that Spanish learners' academic resilience, assessed by the indices of hardiness and resourcefulness, can predict academic achievement. On the other hand, Marie et al. (2021) found a non-significant relationship between pharmacy learners' academic performance in math and their academic resilience. Kim and Kim (2021) found out that academic resilience is related to the various L2 educational issues including learning motivation.

### The Concept of L2 Motivation

The learning process requires motivation, and learners should self-motivate to react appropriately without getting into emotional troubles, such as vulnerability, indifference, despair, or distress (Alvarez-Ramírez and Cáceres, 2010). Csizér and Dörnyei (2005) pointed out that “motivation is a concept that explains why people behave as they do rather than how successful their behavior will be” (p. 20). Nevertheless, Dörnyei (2001) asserted the indirect relationship between motivation and language learning results since learners' proficiency in a foreign language may be affected by their linguistic skills, and linguistic backgrounds. He also maintained that a demotivated individual primarily has had the motivation to engage in an activity for reaching a goal. However, some external negative factors like school and classroom contexts have made them demotivated (Dörnyei, 2001). L2 motivation signifies a multi-layered psychological concept that drives a learner to learn a second or foreign language (Dörnyei, 2005). Alrabai and Moskovsky (2016) showed that motivation, autonomy, viewpoint, anxiety, and self-esteem are critical in L2 learning proficiency. They mentioned that motivation has the highest predictability power over other factors in language learning proficiency. Crookes and Schmidt (1991) highlighted the importance of regarding four motivational components of Keller (1983) educational orientation theory including (a) interest; (b) relevance; (c) expectancy; and (d) satisfaction. They maintained that these components of motivation should be taken into account by instructors when they are trying the appreciate learners' learning motivation since these components may affect learner's language learning (Crookes and Schmidt, 1991).

The integrative and instrumental motivations have been specified as two leading components of L2 learning motivation (Fan and Feng, 2012; Carrió-Pastor and Mestre, 2014; Quan, 2014). Motivation is a blend of internal and external forces that urges learners to focus their attempts on the learning process (Gardner, 2010). Learners' integrative motivation refers to their willingness to integrate themselves into a spoken language society. Moreover, learners' positive viewpoints toward the teaching-learning context determine their integrative and their academic achievement (Khodadad and Kaur, 2016).

Instead, Gardner and Lambert (1972, p. 150) pointed out that “instrumental orientation is considered a desire to gain social recognition or economic advantages through knowledge of a foreign language.” Cheng et al.'s (Cheng et al., 2014) asserted that the higher instrumental motivation leads to the lower test scores. The findings of Lukmani (1972) study showed that Indian EFL learners' instrumental motivation can significantly correlate with language proficiency scores. Overall, Gardner (2001) asserted that some factors such as positive attitudes toward learning context, instrumental and integrative-oriented motivations significantly affect learners' attention, and the desire that will regulate learners' endeavors in language learning. Khalid (2016) found out that instrumental and integrative motivation in language learning is associated with learners' socioeconomic position. He also asserted that attitudes toward language learning can be affected by these orientations. Gardner (1985) pointed out L2 motivation includes three components including motivational intensity, desire to achieve language learning objective and positive attitudes toward language learning and the native speakers of that language. Motivational intensity is going to be discussed in this study.

### Motivational Intensity

Motivational intensity is defined as the consistent efforts of learners during their language learning process. Then, it is considered the goal-oriented effort that learners spend to learn a foreign language and their persistence in education (Ellis, 2004). Ely (1986) also highlighted the significance of studying the power of the learner's motivation or, in other words, his/her motivational intensity, “it also seems important to investigate the strength of that motivation” (p. 28). According to Dörnyei (1998), a learner's motivated behavior can be obviously interpreted as his/her motivational intensity. He pointed out that “the proof of motivation is in displaying it in action—hence the importance of the “desire” measure, which directly taps into the individual's wish to perform the action; and, even more directly, the “motivational intensity” measure that explicitly focuses on motivated behavior” (p. 122). Masgoret and Gardner (2003) indicated that desire and attitude have a significant effect on the learners' motivational intensity.

In order for a language learner to succeed, their motivational intensity has a critical role (Pintrich and Schunk, 2002; Wang and Guan, 2020). Cocca and Cocca (2019) investigated the predictability of affective variables and motivational intensity in language learners' academic achievement and their study revealed the significant relationship between learners' motivational intensity, their attitudes toward learning English and their language proficiency. Mori and Gobel (2021) studied the effect of studying abroad and learners' language ability and their motivational intensity. They found a strong correlation between learners' desire and their motivational intensity. However, their desire to learn and the motivational intensity before the departure have a significant relationship with their language ability measured by TOEIC listening and reading test. Ahmad (2005) stated that gender difference, socio-economic and educational backgrounds are significantly correlated with motivational intensity while classroom anxiety is negatively correlated with motivational intensity in EFL contexts.

### The Relationship Between Motivation and Resilience

According to the theoretical relationships between L2 learning achievement and the individual difference constructs, some investigations have been done on the relationship between L2 learners' motivation and resilience and their significance in language learning (Kim and Lee, 2014; Kim et al., 2017; Xue, 2021). Using a questionnaire to investigate relationships among EFL learners' resilience, motivation, and L2 achievement, Kim and Kim (2016) found out that persistence as resilience factor is significantly related to the learners' motivation and language proficiency. In the same vein, Kim et al. (2017) found out that language learning demotivation is significantly related to language proficiency. Furthermore, their study revealed that resilience, through language learning demotivation, significantly affect language proficiency. Kim et al.'s (Kim et al., 2019) study also revealed that resilience is significantly related to EFL learning motivation. However, they found four factors affecting academic resilience in order to limit the demotivating factors: social support, emotional control, well-defined learning objectives, and perseverance in EFL learning. Their study revealed that learners' emotional control and learning objectives are critical in preserving high academic resilience. Kim and Lee's (Kim and Lee, 2014) study indicated that demotivated EFL learners, in comparison with the motivated ones, revealed low resilience levels. Despite different constructs, motivation plays the role of mediator in the process of being resilient (Resnick et al., 2018).

## Implications and Suggestions

Hiver and Sánchez Solarte (2021) pinpointed that the term “resilience” has drawn the wide attention of investigators in the literature; however, it has not been elucidated. Nonetheless, in recent years, this notion has been accentuated by both investigators and instructors for its potential consequences. The related literature showed that protective factors and risk factors of resilience are effective in learner's academic achievements as they can raise the possibility of a progression or decrease negative consequences. Studies emphasized the importance of social support including peer, community, school, and family support as the variables for academic resilience and they can result in academic achievement. Consequently, policymakers, managers, instructors, and parents should develop these types of strategies to boost learner's academic resilience. This review supported the role of academic resilience in sustaining motivation for language learning in the EFL context. This review may contribute to the prominence of motivational intensity and demotivational factors in the EFL context. The literature review showed some reasons for the lack of resilience and motivation for teachers to recognize which factors hinder learners to accomplish in the classroom contexts. This review implies that teachers can change learners' academic engagement and achievement by paying attention to learners' academic resilience and their motivation. The administrators should enhance EFL contexts in support of learners' motivation by allowing teachers to expand their options in choosing materials, teaching methodology and teaching styles which arouse learners' motivation. Given that the learner achievement is the leading aspect of education, the provision of strategies for increasing learners' resilience and motivation in pre-service and in-service teacher training programs by teacher educators can be useful. Similarly, school managers are recommended to hold educational workshops considering learners' resilience, their motivation and their effects on learners' academic knowledge. They can also provide lectures about the way teachers can increase academic resilience and motivation among learners. Finally, the significance of academic resilience and motivation allow consultants to develop programs to increase the effect of these variables on learning performance. They can identify learners who need some support by arranging them based on level of academic resilience and motivation.

Many studies have been done on academic resilience, however, the changing context and culture made investigators delve into the term meticulously. One of the factors that may influence the learners' resilience is learning context. Moreover, further studies need to be done in order to conduct a comparative study in terms of different study fields and their relationship with learners' academic resilience and their motivational intensity. Similarly, the investigation of resilience among the learners in the EFL context is in dire need of attention and a narrow body of studies have been conducted on this issue. Motivation is primarily a crucial factor for resilience and it also plays an important role in L2 learning. This study mostly reviewed the mediating role of resilience between language learning motivation and language learning achievement. Language learning motivation can be deeply affected by context and L2 language proficiency (Martin, 2020). Owing to the multifaceted nature of the notion of academic resilience, some investigations should be conducted to realize other influencing factors other than academic resilience in academic achievement. Some protective factors of resilience such as emphatic understanding, peer relations, parents' high expectations, teachers' attention and kindness, learner's positive attitudes toward the adversity, self-efficacy, academic engagement, control, anxiety, contributing to in-school activities, communication factors, type of methodology, qualities of school, the size of a class, the financial contribution of parents' to education, gender, learners' geographical area of residence, economic and social class level, the average score for school achievement, parents' educational position, self-control and cognitive flexibility can be studied for the future and their relationship with academic achieving and learners' motivation intensity can be scrutinized in order to help decision-makers in education policy (Wang and Guan, 2020). Global digitalization influenced individuals' life in the educational context and language learning is also affected by this advancement. Online language learning in EFL context, especially in adverse occasions, such as COVID-19 pandemic, severely disturbed language learning (Wang and Derakhshan, 2021). Further studies are required to conduct comparative studies between interpersonal factors such as motivation and academic resilience in traditional and digital situations and elucidate how these occasions might influence language learning and teaching experiences. Other than resilience, the predictability of some positive psychology factors such as enjoyment, grit, well-being, loving pedagogy, self-compassion, engagement, and emotional regulation in academic achievement and motivational intensity can be studied for the future in order to contribute to foreign language learning experience. The investigation of emotional resilience and its relationship with learners' motivational intensity and their academic achievement in different language contexts can also be considered in the future.

## Data Availability Statement

The raw data supporting the conclusions of this article will be made available by the authors, without undue reservation.

## Author Contributions

All authors listed have made a substantial, direct, and intellectual contribution to the work and approved it for publication.

## Funding

This study was sponsored by the project of Hebei Philosophy and Social Science Fund-the interactive mechanism, effect evaluation and improvement strategy of rural revitalization and migrant workers' return to their hometowns in Hebei Province (approval number: HB21RK001).

## Conflict of Interest

The authors declare that the research was conducted in the absence of any commercial or financial relationships that could be construed as a potential conflict of interest.

## Publisher's Note

All claims expressed in this article are solely those of the authors and do not necessarily represent those of their affiliated organizations, or those of the publisher, the editors and the reviewers. Any product that may be evaluated in this article, or claim that may be made by its manufacturer, is not guaranteed or endorsed by the publisher.
